# Brain-Derived Neurotrophic Factor and Antidepressive Effect of Electroconvulsive Therapy: Systematic Review and Meta-Analyses of the Preclinical and Clinical Literature

**DOI:** 10.1371/journal.pone.0141564

**Published:** 2015-11-03

**Authors:** M. Polyakova, M. L. Schroeter, B. M. Elzinga, S. Holiga, P. Schoenknecht, E. R. de Kloet, M. L. Molendijk

**Affiliations:** 1 Max Planck Institute for Human Cognitive and Brain Sciences & Clinic for Cognitive Neurology, University Hospital, Leipzig, Germany; 2 Institute of Psychology, Leiden University and Leiden Institute for Brain and Cognition, Leiden University Medical Center, Leiden, The Netherlands; 3 University Hospital Leipzig, Department of Psychiatry and Psychotherapy, Leipzig, Germany; 4 Division of Medical Pharmacology, Division of Endocrinology, and Leiden Academic Center for Drug Research, Leiden University Medical Center, Leiden, The Netherlands; Peking University, CHINA

## Abstract

Emerging data suggest that Electro-Convulsive Treatment (ECT) may reduce depressive symptoms by increasing the expression of Brain-Derived Neurotrophic Factor (BDNF). Yet, conflicting findings have been reported. For this reason we performed a systematic review and meta-analysis of the preclinical and clinical literature on the association between ECT treatment (ECS in animals) and changes in BDNF concentrations and their effect on behavior. In addition, regional brain expression of BDNF in mouse and human brains were compared using Allen Brain Atlas. ECS, over sham, increased BDNF mRNA and protein in animal brain (effect size [Hedge’s g]: 0.38―0.54; 258 effect-size estimates, *N* = 4,284) but not in serum (*g* = 0.06, 95% *CI* = -0.05―0.17). In humans, plasma but not serum BDNF increased following ECT (*g* = 0.72 vs. *g = 0*.*14;* 23 effect sizes, *n* = 281). The gradient of the BDNF increment in animal brains corresponded to the gradient of the BDNF gene expression according to the Allen brain atlas. Effect-size estimates were larger following more ECT sessions in animals (*r* = 0.37, P < .0001) and in humans (*r* = 0.55; *P* = 0.05). There were some indications that the increase in BDNF expression was associated with behavioral changes in rodents, but not in humans. We conclude that ECS in rodents and ECT in humans increase BDNF concentrations but this is not consistently associated with changes in behavior.

## Introduction

Electro Convulsive Treatment (ECT) has been used as a treatment for mood disorders for years. There is little doubt on the clinical efficacy of ECT [1, 2], yet, how it improves mood remains unclear [3, 4]. Emerging data have led to the idea that ECT may reduce depressive symptoms by increasing the expression of Brain-Derived Neurotrophic Factor (BDNF), a key regulator of neuronal functioning [5]. This idea rests on the *neurotrophin hypothesis*, which posits that depressive disorders are secondary to a stress-induced lowered expression of BDNF [6]. Complementary, it predicts that antidepressants are efficacious, because they increase BDNF expression and herewith boost neuronal plasticity [7–9].

Preclinical and clinical studies both have provided support for the neurotrophin hypothesis. Nibuya *et al*. [10], for instance, showed in rats that Electro-Convulsive Shocks (ECS, the equivalent of ECT in animals) increases the expression of hippocampal BDNF mRNA. This has been replicated and extended to other brain regions (*e*.*g*., the amygdala [11]) and was shown for BDNF protein levels [12]. Interestingly, and in line with the neurotrophin hypothesis, some studies show that the increase in BDNF following ECS is associated with a decrease in depression-like behaviors.

Measurements in brain tissue, as they are applied in preclinical studies, obviously cannot be pursued in humans. Clinical studies usually measure the change in peripheral (*e*.*g*., blood serum) BDNF protein concentrations over treatment with ECT. The validity of this approach is based on the observation that the brain is in part the source of BDNF in peripheral tissues [12, 13]. Clinical studies show that peripheral BDNF concentrations increase following treatment with ECT, as evidenced by a recent a meta-analysis (Hedge’s *g* = 0.38, 11 studies, 221 subjects) [14]. In contrast to some individual preclinical (*e*.*g*., Li *et al*. [15]) and clinical studies (*e*.*g*., Hu *et al*. [16]), this meta-analysis did not find evidence for the notion that changes in BDNF concentrations over treatment are related to the clinical efficacy of ECT. This omission may be due to a limited number of trials and patients and the use of group-level statistics [17]. An additional factor explaining the lack of association may be that serum and plasma BDNF measurement were merged in the analyses. Plasma levels are likely to reflect momentary BDNF protein expression, while serum levels reflect accumulated (over a period of about 10 days) BDNF [18–20]. The combination of plasma and serum measurement in a single meta-analysis, as was done by Brunoni *et al*. [14], therefore may not be biologically plausible.

Notwithstanding some contradictory findings, the data above suggest a relation between ECT treatment and BDNF expression. The goal of this study, then, was to evaluate, through systematic review and meta-analyses, the preclinical (*i*.*e*., rodent) and clinical (*i*.*e*., human) literature on changes in BDNF concentrations and behavior over the course of ECS and ECT respectively. To fulfill this translational aim, we first will pool the preclinical literature on the relationships between ECS, BDNF and depression-like behavior. Next, we will aggregate effect-sizes of ECT treatment on BDNF concentrations and clinical improvement as they are reported in the human literature. This will be done partially using meta-analysis on individual data because this better suits the questions at hand given a limited number of trials and patients that are available [17].

## Materials and Methods

We adhered to the guidelines that are recommended by the preferred reporting items for systematic reviews and meta-analyses statement [21].

### Search Strategy

We searched PUBMED, Embase, and PsychInfo through December 1^st^ 2014 to identify eligible studies on changes in peripheral and central BDNF concentrations as a function of treatment with ECT. The following keywords were used: ‘electroconvulsive’ or ‘ECT’ or ‘ECS’ in combination with ‘BDNF’ or ‘brain derived neurotrophic factor’. This was supplemented by backward searches in which the references to the seminal papers of interest were screened for preclinical and clinical studies and by examining the reference sections of the retrieved papers. The literature search, decisions on inclusion, data extraction, and quality control were performed independently by two of the authors (MP and MM).

### Inclusion and Exclusion Criteria

We included peer-reviewed preclinical and clinical studies that reported data on BDNF concentrations as a function of ECS/ECT (*i*.*e*., ECS/ECT *versus* sham and pre *versus* post treatment). Inclusion was independent of ECS/ECT characteristics (*e*.*g*., number of sessions) and methodological characteristics of the study (*e*.*g*., tissue in which BDNF was sampled). For the clinical studies diagnosis of major- or bipolar depression had to be based on international classifications.

Non-empirical studies such as reviews were excluded according to review protocol, as were case studies, studies that were not peer reviewed, and studies that were not written in Dutch, English, French, German or Spanish. Where study samples overlapped we excluded the study that reported on the fewest number of subjects.

### Data Extraction

From each paper we extracted, as primary outcomes, mean BDNF concentrations (and *Standard Deviation* [*SD*]) in treatment conditions *versus* sham and/or before and after ECS/ECT or indices on this change (*e*.*g*., the standardized mean difference). We also extracted data on mean age, gender distribution, specifics of the ECS/ECT treatment, and the method that was applied to quantify the amount of BDNF (*e*.*g*., RT-PCR).

From the preclinical studies we further extracted data on the strain of animal that was used, the weight and age of the animals, the brain-region in which BDNF was measured, and the amount of time between ECS treatment and decapitation. Data on behavioral changes due to ECS were extracted where provided.

From the clinical studies we in addition extracted data on depression severity pre- and post ECT, whether participants exhibited a clinical response to ECT, the antidepressant that were used, and the amount of time between the last ECT session and blood draw for BDNF determination. In order to perform subgroup comparisons according to treatment response we contacted the authors of the clinical studies and asked them to provide anonymised Individual Patients Data (IPD) [17]. In those cases where non-significant results were reported (*e*.*g*., *P*>.05) and authors did not reply to our request for exact outcome data, we set the association at *P* = .50, indicating no association.

We assessed the methodological quality of the preclinical and clinical studies using the ARRIVE guidelines[22] and the Newcastle-Ottawa Scale (NOS) [23] respectively. In addition we used the risk of bias assessment tool for the longitudinal studies [24]. We refer to the Supplement for detailed information on quality assessment (S1 Text, S1 Table, S2 Table, S3 Table).

### Statistical Analysis

Analyses were performed using Comprehensive Meta-Analyses 2.0 [25] and SPSS version 21.0 [26].Random effects models (*i*.*e*., models that include sampling- and study level error) were applied to calculate pooled effect-sizes on changes in central and peripheral BDNF concentrations as a function of ECS/ECT. As effect-size measure we chose to use Hedges’ *g*, a standardized metric that corrects for bias related to small sample sizes [27]. All outcomes were weighted using inverse variance methods [25]. Statistical significance was assessed using a *Z*-statistic at a Confidence Interval (*CI*) of 95%. The amount of between-study heterogeneity in outcomes was quantified using the *I* ^2^ measure [28] and assessed for statistical significance using the *Q*-statistic[25].

The stability of our results was evaluated through meta-analyses that were run in specific subgroups: (**I**) by brain region in which BDNF was assessed (clustered as follows: Dentate Gyrus [DG], hippocampus not DG, cortex, other brain regions, and in serum [S4 Table], (**II**) single *versus* multiple ECT sessions, and (**III**) the type of BDNF that was measured (*i*.*e*., BDNF mRNA *versus* protein and BDNF in serum *versus* in plasma). The possible moderating effects of between-study differences on outcomes were evaluated by calculating correlation coefficients between the values for the moderator and the outcome of the studies.

For the analyses on preclinical data, the animal strain that was used, duration of treatment, the amount of time between the last ECT session and decapitation for BDNF measurements, and the quality score were considered as potential moderators. For clinical data analysis, obtained IPD were combined with the aggregated data using a two-step approach. In a first step summary statistics were calculated for each subgroup from single studies. In the second step summary statistics from the IPD were combined in meta-analysis as described above. Treatment response was considered as reduction of depression severity scores by ≥50%. Duration of treatment and the quality score were considered as potential moderators of the effect-sizes retrieved from clinical studies.

Visual inspection of funnel plots and the Egger test were used to assess publication bias [29]. In case of publication bias we used trim-and-fill procedures to estimate effect-sizes after bias has been taken into account [30].

## Results

### Preclinical Studies

**Fig 1:**
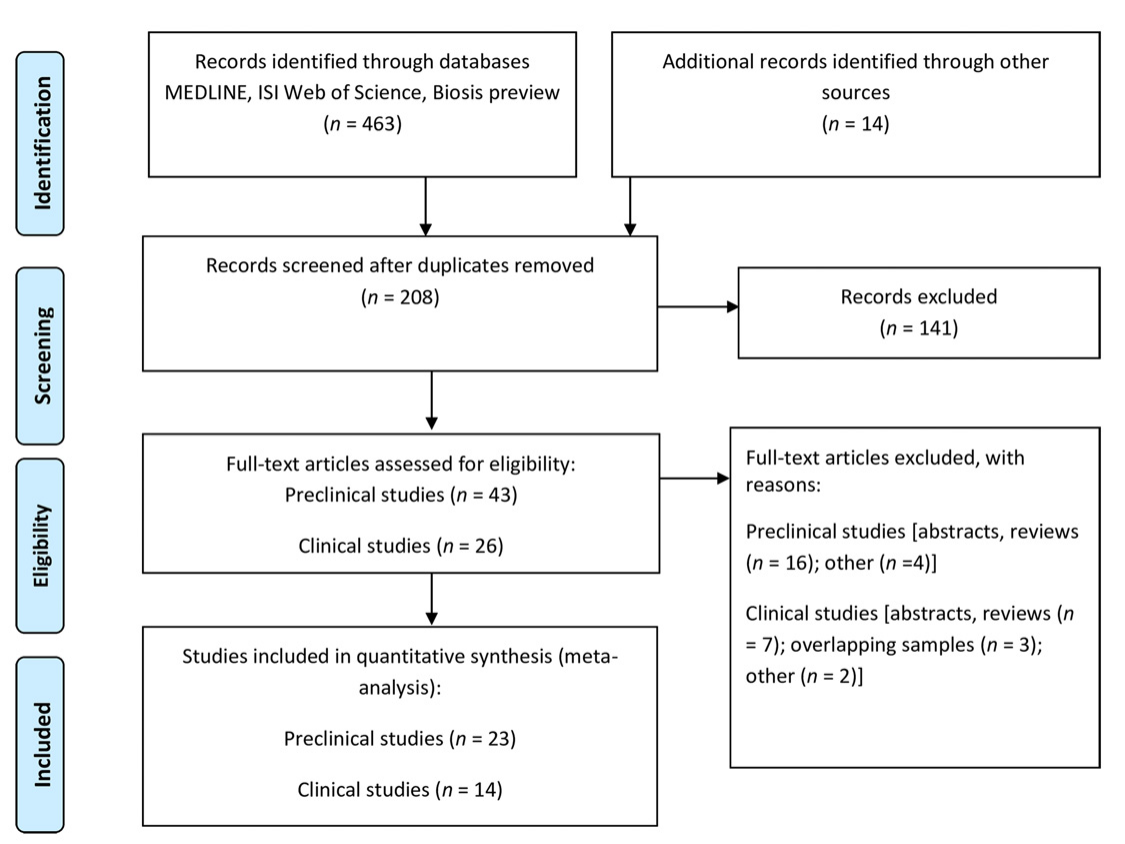
Prisma flow diagram of the search strategy and its results.

Our search generated 97 papers of which 23 [10–11,15,33–51] fulfilled the inclusion criteria (see Fig 1 for a flow-chart). From these we could extract 280 effect-size estimates (*k*) on a total of 4,670 animals (mean *n* = 17 per effect-size, range 8–30) on changes in BDNF concentrations in animals that were subjected to ECS as compared to sham treatment or, in one case, to baseline.[31] Mean number of ECS sessions was 5 (range: 1–14). Mean time that passed between last ECS session and decapitation was 40 hours (range: 1–504 hours). We refer to Table 1 for the included studies and general information on them. S5 Table and S6 Table provide additional information on the animals that were used and the methods that were applied.

**Table 1:** Basic information on the preclinical studies included in the meta-analysis.

Study	Animal ^A^	ECT	*n* ^B^
Lindefors *et al*.[32]	Sprague-Dawley rats	*Single*: 1 p/d for 1 d	6
Nibuya *et al*.[10]	Sprague-Dawley rats	*Single*: 1 p/d for 1 d and *Multiple*: 1 p/ for 10 d	8
Zetterström *et al*.[33]	Sprague-Dawley rats	*Single*: 1 p/d for 1 d and *Multiple*: 5 over 10 d	5
Chen *et al*.[34]	Sprague-Dawley rats	*Multiple*: 1 p/d for 10 d	6
Altar *et al*. [11]	Wistar rats	*Single*: 1 p/d for1, 2 and 3 d and *Multiple*: 1 p/d for 4, 6, 10 d	7–9
Angelucci *et al*.[35]	FRL and FSL rats	*Multiple*: 1 p/d for 10 d	7
Dias *et al*.[36]	Sprague-Dawley rats	*Single*: 1 p/d for1, 2 and 3 d, and *Multiple*: 1 p/d for 10 d	5
Newton *et al*. [37]	Sprague-Dawley rats	*Single*: 1 p/d for1 d and *Multiple*: 1 p/d for 10 d	5
Jacobsen *et al*.[38]	Wistar rats	*Multiple*: 1 p/d for 10 d	8
Li *et al*.[39]	Wistar rats	*Multiple*: 6 or 14 for 6 or 14 d	15
Ploski *et al*. [40]	Sprague-Dawley rats	*Multiple*: 1 p/d for 14 d	8
Conti *et al*. [41]	Sprague-Dawley rats	*Multiple*: 8 for 2 d	8
Li *et al*. [15]	Wistar rats	*Multiple*: 14 for 14 d	7–8
Sartorius *et al*.[31]	Sprague-Dawley rats	*Single*: 1 p/d for1 d and *Multiple*: 1 p/d for 5 d	8
Gersner*et al*. [42]	Sprague-Dawley rats	*Multiple*: 1 p/d for 6–14 d	10
Kyeremanteng *et al*.[43]	Wistar-Kyoto rats, Wistar rats	*Multiple*: 5 p/d for 5 d	10
Luo *et al*.[44]	Wistar rats	*Multiple*: 1 p/d for 6–14 d	10
O'Donovan *et al*.[45]	Sprague-Dawley rats	*Multiple*: 10 sessions over 3–4 weeks	8
Ryan *et al*.[46]	Sprague-Dawley rats	*Single*: 1 p/d for 1 d and *Multiple*: 10 sessions over 21–28 d	8
Segawa *et al*. [47]	Sprague-Dawley rats	*Single*: 1 p/d for1 d and *Multiple*: 1 p/d for 10 d	8
Segi-Nishida *et al*.[48]	C57BL/6N mice	*Single*: 1 p/d for 1 d and *Multiple*: 1 p/d for 6 and for 14 d	4
Dyrvig *et al*. [49]	Sprague-Dawley rats	*Single*: 1 p/d for 1 d	6
Kyeremanteng *et al*.[50]	Wistar-Kyoto rats, Wistar rats	*Multiple*: 5 p/d for 5 d	9–10

^A^ all studies assessed male animals. Sartorius *et al*.[31] and Gersner *et al*.[51] did not specify the sex of the animals they used.

^B^ *n* is given per group and, in general can be doubled for the experimental vs sham comparison.

All studies applied sham ECT as the control condition, except for the study by Sartorius *et al*.[31] in which *baseline* was considered as the control condition.

In the S5 Table we present additional information on the included preclinical studies (*e*.*g*., age and weight of the animals).

### Meta-analysis over preclinical findings

**Fig 2:**
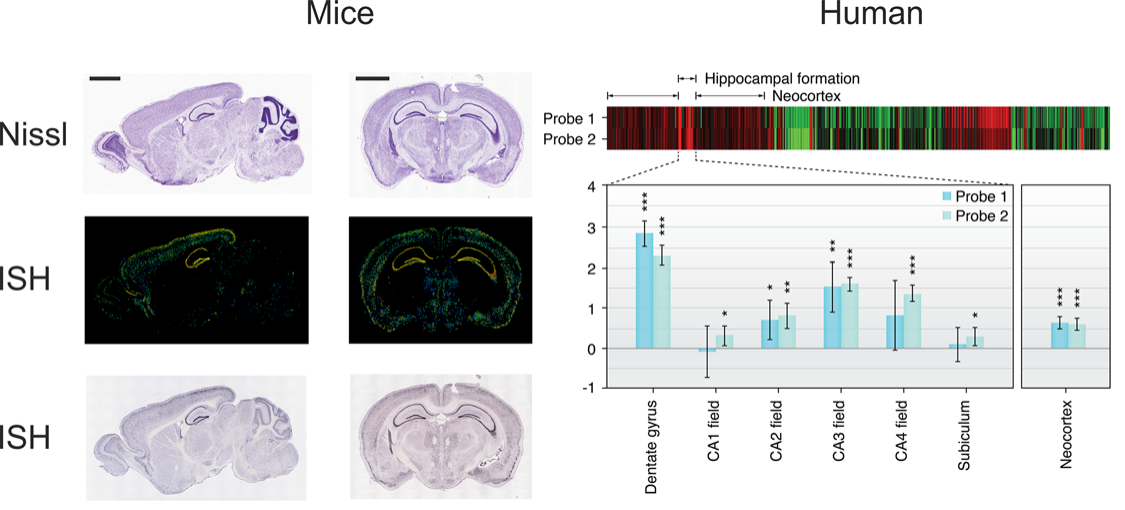
Gene expression of BDNF across the whole brain as assessed in Allen Brain Atlas (Seattle, WA, USA). In mice, gene expression was the highest in the DG/hippocampus as investigated by In Situ Hybridization (ISH), where warm colors indicate high expression. Note that contrast and brightness were enhanced in original images to increase visibility of the effects here. Black bars correspond to 2 mm. Gene expression in humans is shown as individually normalized gene expression (*Z*-scores normalized to whole human brain expression). The heat map shows scores across the whole human brain and for each of the six subjects contained in the database beside each other, where red indicates high and green indicates low expression. Bars represent mean normalized gene expression and standard deviation across one female and five male subjects included. Search conducted on 17^th^ October 2014 for human and on 9^th^ October 2014 for mice data. *** *P* < 0.001; ** *P* < 0.01; * *P* < 0.05, two-tailed Student’s *t*-test against 0. For details on the methods we refer to Mueller *et al*.[53]

ECS was associated with increased BDNF concentrations in comparison to sham treatment (*g* = 0.40, 95% *CI* = 0.35―0.44, *P* < .0001; 280 effect-sizes, *N* = 4,284). Meta-analyses by specific brain region showed a larger effect-size (*P* < .05) when BDNF was assessed in the DG (*g* = 0.54) as compared to assessments in the hippocampus and the cortex (*g*: 0.38―0.41 respectively). Yet, effect-sizes were significant regardless in which brain area BDNF was sampled (see **Table 2**). Interestingly, the observed gradient of ECS induced increases in BDNF protein corresponds to the gradient of BDNF gene expression across the whole brain in mice and humans as assessed in the genome wide atlas of the Allen Institute for Brain Sciences (Seattle, WA, USA, see www.brain-map.org) [52]. Results of this analysis are illustrated in **Fig 2** with highest gene expression in DG, followed by hippocampus and other brain regions.

**Table 2:** Pooled effect-size estimates, heterogeneity and publication bias for the animal studies by sub-group meta-analyses indicated in the row.

	*k*	*N*	Hedges’ *g* (95% *CI*)	Heterogeneity		Publication bias
				*I* ^2^	*Q*	Egger’s *t*
**BDNF sampled in:** ^**A**^						
DG	25	384	0.54 (0.42–0.67) ***	23.6%	31.4	3.1 *
Hippocampus	124	2,032	0.38 (0.32–0.45) ***	49.3%	242.8 ***	5.2 ***
Cortex	57	982	0.41 (0.32–0.51) ***	51.5%	115.6 ***	3.3 **
Other	61	976	0.44 (0.34–0.54) ***	56.8%	138.9 ***	6.9 **
Serum	13	296	0.06 (-0.05–0.17)	0.1%	6.7	0.3
**Number of sessions:** ^**B**^							
Single treatment	78	1,282	0.22 (0.12–0.29) ***	44.3%	138.3 ***	5.5 **
Multiple treatment	202	3,388	0.46 (0.38–0.48) ***	49.6%	398.9 ***	8.8 **
**BDNF type:** ^**C**^							
BDNF protein	147	2,795	0.35 (0.29–0.41) ***	49.0%	286.5 ***	8.2 **
BDNF mRNA ^**D**^	133	1,875	0.46 (0.39–0.53) ***	51.0%	224.8 ***	7.5 **

^**A**^ Effect-size estimates were of a larger magnitude in studies that measured central- as compared to serum BDNF (all *P*-values < .001). Furthermore, larger effect-size estimates were found in the DG as compared to those found in the hippocampus and the cortex (*P*-values < .05). There were no statistically significant differences in pooled effect-size estimates derived from the hippocampus, the cortex and other brain regions (all *P*-values > .5).

^**B**^ Chronic ECS yielded larger effect-size estimates as compared to single ECS (*P* < .0001).

^**C**^ Studies that sampled BDNF mRNA yielded larger effect-size estimates as compared to studies that sampled BDNF protein (*P* < .01).

^**D**^ Among the studies that are characterized as measuring BDNF mRNA were 3 effect-sizes on BDNF RNA and 9 on the precursor protein pro-BDNF. Excluding these effect-sizes did not change the results.

* Statistical significant at *P* < .05

** Statistical significance at *P* < .01

*** Statistical significance at *P* < .001.

Evidence for increases in serum BDNF concentrations (*i*.*e*., in blood serum) following ECS was not found in the preclinical data (*g* = 0.06, 95% *CI* = -0.05―0.17). In fact, the pooled effect-size on serum measurement was smaller as compared to the ones calculated on central BDNF (*P*-values all < .001). Studies that subjected animals to multiple ECS’s yielded larger effect-size as compared to studies that applied single ECS (*P* < .0001). Yet, also a single ECS session was associated with an increase in BDNF (see **Table 2**). The pooled effect size that was derived from studies that measured BDNF mRNA was larger as the one from studies that measured BDNF protein (*P* < .0001) although the latter also was statistically significant (see **Table 2**).

Between-study heterogeneity in outcomes was identified (*I* ^2^ = 51%, *Q* = 572.13, *P* < .00001). The number of ECS sessions that was applied and the time that passed between the last ECS session and measurement appeared to be sources of the observed heterogeneity. A larger number of treatment sessions, in general was associated with larger effect size estimates (*r* = 0.36, *R* ^*2*^ = 0.13, *P* < .0001) and a longer gap in time between the last ECS session and decapitation with smaller effect-size-estimates (*r* = -0.27, *R* ^*2*^ = 0.07, *P* < .0001). The correlation between the number of ECS and ECS induced increase in BDNF was also present within the multiple treated animals (*r* = 0.35, *R* ^*2*^ = 0.13, [202 data points], *P* < .0001). The methodological quality of a study was unrelated to outcome.

The funnel plot and the Egger’s test suggested evidence for publication bias in the overall analysis (*t*_[278]_ = 10.41, *P* < .0001). Imputation of 15, presumed missing, effect-size estimates resulted in a symmetric funnel-plot. The pooled effect-size estimate that was recalculated after imputation was only slightly smaller as compared to the one derived in the original analysis (*g* = 0.38, 95% *CI* = 0.35―0.41). Between-study heterogeneity, correlations between outcomes and moderators, and publication bias in outcomes were rather similar in analyses that were run separately in the subgroups (see **Table 3**).

**Table 3:** Pearson’s correlation coefficients on the relation between study characteristics and study effect size (by meta-analysis indicated in the columns).

	All	DG	Hippocampus	Cortex	Other
**BDNF mRNA and protein**	*k* = 267, *n* = 4,374	*k* = 25, *n* = 384	*k* = 124, *n* = 2,032	*k* = 57, *n* = 982	*k* = 61, *n* = 976
Number of treatments ^A^	0.36***	0.10	0.43***	0.46***	0.28*
Time of measurement after last ECT	-0.22***	-0.38	-0.17	-0.30*	-0.35**
**BDNF mRNA**	*k* = 133, *n* = 1,489	*k* = 25, *n* = 384	*k* = 65, *n* = 933	*k* = 17, *n* = 222	*k* = 26, *n* = 336
Number of treatments	0.29**	0.10	0.29*	0.41	0.20
Time of measurement after last ECT	-0.39**	-0.38	-0.24	-0.29	-0.38
**BDNF protein**	*k* = 147, *n* = 2,795	*k* = 0, *n* = 0	*k* = 59, *n* = 1,099	*k* = 40, *n* = 744	*k* = 35, *n* = 640
Number of treatments	0.48***	*NA*	0.61***	0.54***	0.25
Time of measurement after last ECT	-0.21*	*NA*	-0.10	-0.32*	-0.33*

^**A**^ The correlation between number of treatments and outcome was also present in studies that applied multiple treatments (*r* = .35 (202 data points) *P* < .0001).

Abbreviation. *NA*; Not Applicable.

* Statistically significant at *P* < .05

** statistically significant at *P* < .01

*** statistically significant at *P* < .001.

**NOTE.** There was no evidence for between-study heterogeneity in the meta-analyses on serum BDNF levels. Correlational analyses therefore were not performed in this sub-group.

#### ECS, BDNF and changes in behaviour

There was too little comparable data on behavioral tests (*e*.*g*., the open-field test) to perform meta-analysis on. In case similar behavioral paradigms were applied, often the outcome measures over studies were different. This was for instance so for swimming time in the FST for which we could extract 48 effect-size estimates (910 animals, range: 11―30 per effect-size) on total swimming time in the FST. Together, these showed that ECS, over sham, increased swimming time (*g* = 0.26, 95% *CI* = 0.19―0.32, *P* < .0001). The increase in swimming time correlated positively with the increase in BDNF (*r* = 0.37, *R* ^*2*^ = 0.14, *P* < .001). Note though that these effect sizes came from only four studies that widely differed in for instance in time of sacrifice after ECS and other variables that potentially can confound the observed relation. The association, thus, should be interpreted with caution.

#### Sensitivity analyses

None of the study findings was unduly driven by the effect of a particular study (data not shown). Furthermore, effect-size estimates were not related (*P* = .49) to whether or a particular study used a stress paradigm (*e*.*g*., chronic unpredictable mild stress). Method of BDNF measurement was not associated with the amount of change in detectable BDNF (*P* = .17; see **S6 Table** for the methods of measurement by study). Animal strain was tested as a potential effect modifier (see **S6 Table** for the animal strain that was used in each individual study). Analyses showed that there were no differences in ECS induced increases in BDNF as a function of strain of animal that was used in the experiment (*P* = .18).

### Clinical Studies

Our search for clinical studies generated 111 publications of which 14 fulfilled the inclusion criteria (see **Fig 1** for a flow-chart). From these papers we obtained 23 effect-size estimates on changes in BDNF concentrations over the course of ECT (*N* = 250 subjects a [mean *n* = 13 per effect-size, range 3–48]). Ten studies [16, 54–62] reported on serum BDNF alternations (16 effect sizes) and 4 studies [63–66] (7 effect sizes) on plasma BDNF alterations. **Table 4** and **S7 Table** provide details of the included studies.

**Table 4:** Basic information on the clinical studies included in the meta-analysis.

Study	Diagnosis	Source	Response	*N* (f/m)	BDNF levels				
					Pre-treatment		Post-treatment		Unit
					mean	SD	mean	SD	
Bocchio-Chiavetto *et al*.[54]	MDD	serum	Yes	20 (14/6)	27.10	9.31	27.95	8.03	ng/ml
			No	3 (2/1)	31.2	8.42	31.2	8.3	pg/ml
Marano *et al*.[63]	MDD, BD	plasma	Yes	13 (3/10)	83.1	63.0	202.5	179.1	pg/ml
			No	2 (1/1)	119.5	33.3	265.5	236.6	pg/ml
Okamoto *et al*.[55]	MDD, BD	serum	Yes	12 (6/6)	7.9	9.9	15.1	11.1	ng/ml
			No	6 (3/3)	11.5	11.0	9.4	7.5	ng/ml
Fernandes *et al*.[56]	MDD, BD	serum	Yes (73.33%)	15 (10/5)	0.3	0.1	0.3	0.1	pg/ml
Gronli *et al*.[57]	MDD, BD	serum	Yes	10 (NA)	1242.5	187.0	1395.7	517.7	pg/ml
Piccinni *et al*. [64]	MDD, BD	plasma	Yes	8 (5/3)	2.9	1.3	5	1.8	ng/ml
			No	10 (4/6)	1.5	0.5	2.7	1.4	ng/ml
Hu *et al*.[16]	MDD	serum	Yes	24 (20/4)	5.5	1.9	8.08	3.5	ng/ml
			No	4 (3/1)	6.5	3.4	6.9	3.1	ng/ml
Gedge *et al*.[58]	MDD	serum	Yes	5 (2/3)	13.3	6.7	12.4	4.3	ng/ml
			No	6 (5/1)	7.2	5.2	12.2	3.1	ng/ml
Haghighi *et al*.[65]	MDD	plasma	Yes (75%)	20 (5/15)	151.0	174.7	376.7	299.3	pg/ml
Lin *et al*.[66]	MDD, BD	plasma	Yes	48 (38/10)	3652.8	2372.6	3512.6	2104.9	pg/ml
	MDD, BD		No	7 (6/1)	3085.3	2005.6	4190.7	1917.9	pg/ml
Stelzhammer *et al*.[59]	MDD	serum	Yes	3 (3/0)	20.4	13.5	8.2	4.5	ng/ml
			No	4 (2/2)	22.7	7.01	14.3	5.4	ng/ml
Bilgen *et al*.[60]	MDD	serum	Yes	30 (19/11)	1990.5	510	2713.3	382.8	pg/ml
Bumb *et al*.[61]	MDD	serum		20 (10/10)	2596.7	1101.5	3001.8	1118.5	pg/ml
Kleimann *et al*.[62]	MDD	serum	Yes	6	541.2	294.9	342.8	134.4	pg/ml
			No	5	721.8	364.1	506.3	142.0	pg/ml

Abbreviations: MDD, major depressive disorder; BD, bipolar disorder

#### Meta-analysis over clinical findings

Overall peripheral BDNF was significantly increased after ECT as compared to baseline (*g* = 0.35, 95% *CI* = 0.034–0.67, *P* = 0.03; 23 effect sizes, *n* = 281). BDNF levels increased in plasma (*g* = 0.72, 95% *CI* = 0.22–1.23, *P* = 0.004; 7 effect sizes, *n* = 108) but not in serum (*g* = 0.14, 95% *CI* = -0.29–0.56, *P* = 0.67; 16 effect sizes, *n* = 173). However, the difference between serum and plasma subgroups did not rich the significance threshold (*P* = 0.10; **Table 5**). When subdivided into responders and non-responders subgroups, BDNF increased non-significantly in both the responders- (*g* = 0.40 95% *CI* = 0.02–0.82, *P* = 0.06; 13 effect sizes, *n* = 214) and non-responders subgroups (*g* = 0.22 95% *CI* = -0.38–0.82, *P* = 0.48; 9 effect sizes, *n* = 47). There was no different pattern of results when comparing the pooled effect sizes from studies that measured BDNF in serum versus plasma (**Table 5**). However, significant differences could be observed between plasma and serum BDNF in the non-responders subgroups (*P* = 0.05).

**Table 5:** Pooled effect-size estimates, heterogeneity and publication bias for the clinical studies by sub-group meta-analyses indicated in the row.

	*k*	*n*	Hedges’ *g* (95% *CI*)	Heterogeneity		Publication bias
BDNF in serum and plasma ^A^				*I* ^2^	*Q*	Egger’s *t*
Responders to ECT	13	214	0.40 (0.02–0.82) *	75.6%	44.8 ***	0.7
Non-responders to ECT	9	47	0.22 (-0.38–0.82)	40.5%	14.5	1.1
Overall	23	281	0.37 (0.034–0.67) *	65.1%	63.1 ***	1.1
**BDNF in plasma** ^A^						
Responders to ECT	4	89	0.66 (0.06–1.26) *	74.7%	11.9 **	4.0
Non-responders to ECT	3	19	0.87 (-0.04–1.78)	0.0%	0.63	0.2
Overall	7	108	0.72 (0.22–1.23) **	57.9%	14.3*	2.5
**BDNF in serum** ^A^						
Responders to ECT	9	125	0.22 (-0.36–0.80)	78.4%	37.0***	2.7*
Non-responders to ECT	6	28	-0.13 (-0.94–0.68)	35.8%	7.8	0.7
Overall	16	173	0.14 (-0.29–0.56)	69.1%	48.6***	3.1**

^A^ Effect size estimates were medium and significant in studies that measured BDNF in responders subgroup and non-significant in non-responders subgroup. However, there were no statistically significant differences in pooled effect-size estimates between the responders and non-responders subgroups (all P-values > .5).

* Statistical significance at P < .05

** Statistical significance at P < .01

*** Statistical significance at P < .001

Sensitivity analysis showed that the results were not substantially affected by a single study. We observed overall high heterogeneity in outcomes between the studies (Q = 63.11_[_ _22__]_ *P*<0.001, *I* ^*2*^ = 65.14%). This appeared to be driven by the responder subgroups in both serum and plasma (see **Table 5**). In line with the rodent findings, the number of ECT treatments was positively correlated with the effect sizes in combined serum and plasma subgroup (*r* = 0.55; *P* = 0.05). The number of subjects and methodological quality of the study was not associated with outcomes (data not shown).

Publication bias was detected in the serum subgroup and induced by the studies by Stelzhammer *et al. [59]* and Kleimann *et al. [62],* two negative studies with particularly low power. Correction for publication bias by Trim-and-Fill procedure led to an increased effect size (*g* = 0.57, 95% *CI* = 0.11―1.04; 11 effect sizes, *n* = 101). Overall, and in the plasma subgroup, no publication bias was detected.

## Discussion

Our systematic review and meta-analyses investigated changes in BDNF concentrations as a function of ECS and ECT. Our main findings are: (**A**) in rodents, ECS increases BDNF mRNA and protein concentration (or synthesis/release) in the brain, with largest effect sizes measured in the DG, (**B**) the increase in BDNF is positively correlated with number of treatments and negatively with the time between the last ECT and BDNF measurement, (**C**) BDNF concentrations do not increase in the course of treatment in rodent and human serum, yet they increased in human plasma, and (**D**) the increase in BDNF following ECT is also related to the number of treatment sessions but not to clinical outcome in human studies.

In preclinical studies ECS increased BDNF secretion throughout the brain. Activation of distinct promoters of the BDNF gene is responsible for a differently regulated BDNF expression over brain regions [67, 68]. Four out of nine possible BDNF transcripts are expressed in the rat brain [69]. While in most brain regions one or two transcripts are produced, all four are activated in the DG following ECT [36]. Interestingly, BDNF expression as elicited by ECS appeared to be highest in the DG. This relates well to what the Allen brain atlas shows: BDNF expression in the DG of mice and human brains is highest in this region. A constant supply of BDNF here is not restricted to effects of ECS. This may serve neurogenesis, as the DG is one of the main sources of progenitor cells [70, 71].

The effect of single ECS on BDNF concentrations seems to be short-lived (6–8 hours [10, 49]) and does not involve a hard reset after which BDNF expression remains at a constant higher level. The effect of multiple ECS lasted longer as compared to single treatment: up to 14 days post-ECS [15, 31, 39, 45]. On the meta-analytical level this was reflected by a positive correlation between number of treatments and BDNF levels in rodents, and a trend towards such an association (*P* = .06) in humans.

Interestingly, effect sizes were larger for BDNF mRNA as compared to protein concentrations. Several posttranscriptional mechanisms can be responsible for this. First, protein synthesis may be inhibited by a specific class of microRNA molecules, that bind target mRNA and induce its degradation. Several microRNAs are associated with BDNF depletion [72, 73], one of them, microRNA-212, was increased after ECS in rat’s DG [46]. Second, there is evidence of activity-dependent mRNA trafficking of BDNF to dendrites, where it can be stored and translated on demand [74]. Third, an increased BDNF turnover after ECS was proposed[38] and makes sense in light of findings of neurogenesis after ECT [71, 75].

Once BDNF is synthesized it can act locally, be transferred to neighboring brain areas through axonal anterograde transport or secreted to the blood stream. The later property allowed scientists to make inferences about central BDNF secretion from peripheral measurements. However, initial findings of a high positive correlation between central and serum BDNF [76] were not confirmed [77, 78] or at least depended on animal strain and brain region [31]. Neither a correlation between CSF and serum BDNF in humans was demonstrated [79]. In rodents we demonstrated increments in brain but not in the serum BDNF levels.

In clinical studies, ECT increased peripheral BDNF levels with a small to moderate effect size (*g* = 0.35). Compared to a previous meta-analysis on this topic [14], we included newly published studies, obtained individual patient data and took the source of BDNF (*i*.*e*., plasma versus serum) into account. This approach revealed significantly enhanced BDNF after ECT in plasma and not in serum.

Although both plasma and serum BDNF levels are decreased in acute major and bipolar depression [24, 80], they seem to restore differently following antidepressant treatment [24]. The difference between responders and non-responders that we observed in serum BDNF after pharmacological antidepressant treatment was not demonstrated after ECT. Neither did we observe an increase in serum BDNF after ECT. This differs for plasma measurements, where ECT seems to lead to an increase of BDNF but antidepressant treatment did not [24]. Such difference may point to different mechanisms of action of ECT and antidepressants on BDNF synthesis and release.

### Limitations

Our study has a number of limitations. First, obviously we could not match the preclinical and clinical studies according to depressive state, only (roughly) according to the treatment applied. Most of the animal studies used healthy male animals and did not account for the effects of sex and disease on BDNF. The clinical studies, in turn, included both sexes and were based on treatment-resistant depression cases. Furthermore, none of these studies controlled for relevant confounders in longitudinal studies assessing BDNF, such as seasonality [81]. Plasma BDNF studies could be further confounded by measurement errors [24]. Secondly, due to limited data we had to combine treatment effects on major- and bipolar depression even though imaging studies show differential response to ECT for these two groups [82]. Thirdly, most of the clinical studies included antidepressants and ECT premedication which may have affected BDNF concentrations. Fourthly, while meta-analysis of preclinical data had enough power and showed small to medium heterogeneity, the meta-analysis of clinical data was underpowered and showed signs of publication bias. Due to the limited power we could not control for the impact of ELISA kit manufacturer on effect sizes. Finally, effect-size estimates for the preclinical data may have been suboptimal in terms of precision because they were often estimated based on *P*-value and *N*.

## Conclusions

Despite the limitations, animal and human studies seem to complement each other with regard to effects of ECT on BDNF: ECT increases brain BDNF in animals and plasma BDNF in humans. In animals regional BDNF increments after ECT (*i*.*e*., the DG) corresponded to areas with distinct expression shown in the Allen brain atlas. Besides, multiple treatments as compared to single ECT were associated with a larger increase in BDNF in both animals and humans, which is suggestive for a dose-response effect of ECS on BDNF.

### Future Directions

The questions that remain unsolved are: (1) why plasma but not serum BDNF increased in human studies, (2) what is the relationship between BDNF and behavior, and (3) are increments in BDNF after ECT/ECS related to neurogenesis?

The potential differences between serum and plasma may arise from several aspects. Firstly, plasma BDNF levels reflect momentary BDNF content whereas serum levels reflect BDNF that has been accumulated over several days or even weeks [18 – 20]. Secondly, plasma measurements are very sensitive to the laboratory conditions and, thus, error prone [24]. Future studies (following strict methodological recommendations) should clarify whether plasma increment is not an artifact and further investigate the nature of plasma and serum BDNF.

A larger number of studies is needed to understand the relation of behavioral outcomes to BDNF levels. For clinical studies such outcome measurement is well established: response to treatment or clinical remission. Preclinical studies, however, reported rather different, in terms of timeframe and behavioral assessment, data. Therefore, for the later at least partial overlap in outcome variables with previous studies is needed.

Though the behavioral data is still mixed, neurogenesis is required to achieve antidepressive effect of ECS [83]. Survival of newborn neurons is supported by BDNF [84]. The causality and the dose-response relationships between ECS, BDNF, neurogenesis and behavior are the next questions to adress. Moderators of BDNF functioning, most notably the common genetic variant val66met that has been associated with activity dependent BDNF expression [85], might be considered. Relating variation at this locus to hippocampal morphology [86] and functioning [87] however has thus far led to mixed results.

## Supporting Information

S1 Table Quality of the included preclinical studies. (DOCX)

S2 Table Quality of the included clinical studies as measured with the Newcastle-Ottawa Scale. (DOCX)

S3 Table Quality of the included clinical studies as measured with the RBLS. (DOCX)

S4 Table Brain regions in which BDNF was sampled in the preclinical studies that we included. (DOCX)

S5 Table Basic information on the animals that were used in the preclinical studies that were included in our meta-analysis. (DOCX)

S6 Table Basic methodological information on the preclinical studies that were included in our meta-analysis. (DOCX)

S7 Table Basic information on the patients that were included in the clinical studies that were included in our meta-analysis. (DOCX)

S8 Table PRISMA checklist. (PDF)

S1 Text Quality assessment of the included studies. (DOCX)
