# Supplementary material for: Brain-Derived Neurotrophic Factor and Antidepressive Effect of Electroconvulsive Therapy: Systematic Review and Meta-Analyses of the Preclinical and Clinical Literature
Source: PLoS One. 2015 Nov 3;10(11):e0141564. doi: 10.1371/journal.pone.0141564 (PMC4631320; doi:10.1371/journal.pone.0141564)
Supplement: S1 Table — (DOCX) [file pone.0141564.s001.docx]

| **S1 Table.** Quality of the included preclinical studies | | | | | |
| --- | --- | --- | --- | --- | --- |
| **Between-Group Studies** | **Introduction** [3 max] | **Methods** [21 max] | **Results** [6 max] | **Discussion** [5 max] | **Total** [35 max] |
| Lindefors *et al.* (1995) | 1.5 | 8.0 | 5.0 | 3.0 | 17.5 |
| Nibuya *et al.* (1995) | 2.0 | 10.5 | 4.5 | 2.0 | 19.0 |
| Zetterström *et al.* (1998) | 2.0 | 6.0 | 5.0 | 1.0 | 14.0 |
| Chen *et al.* (2001) | 3.0 | 10.0 | 5.0 | 3.0 | 21.0 |
| Altar *et al.* (2003) | 2.0 | 15.0 | 5.0 | 2.0 | 24.0 |
| Angelucci *et al.* (2003) | 2.0 | 14.0 | 4.0 | 3.0 | 23.0 |
| Dias *et al.* (2003) | 3.0 | 11.0 | 5.0 | 3.0 | 22.0 |
| Newton *et al.* (2003) | 2.0 | 11.0 | 5.0 | 2.0 | 20.0 |
| Jacobsen *et al.* (2004) | 2.0 | 12.0 | 5.0 | 3.0 | 22.0 |
| Li *et al.* (2006) | 3.0 | 9.0 | 5.0 | 2.0 | 19.0 |
| Ploski *et al.* (2006) | 2.0 | 8.0 | 5.0 | 3.0 | 18.0 |
| Conti *et al.* (2007) | 2.0 | 8.0 | 4.0 | 3.5 | 17.5 |
| Li *et al.* (2007) | 3.0 | 11.0 | 5.0 | 3.0 | 22.0 |
| Sartorius *et al.* (2009) | 3.0 | 10.0 | 4.0 | 3.0 | 20.0 |
| Gersner *et al.* (2010) | 2.0 | 11.0 | 4.0 | 2.0 | 19.0 |
| Kyeremanteng *et al.* (2012) | 2.0 | 15.0 | 4.0 | 3.0 | 24.0 |
| Luo *et al.* (2012) | 2.5 | 13.0 | 5.0 | 2.5 | 23.0 |
| O’Donovan *et al.* (2012) | 3.0 | 14.0 | 5.0 | 3.0 | 25.0 |
| Ryan *et al.* (2013) | 1.0 | 8.0 | 5.0 | 3.0 | 17.0 |
| Segawa *et al.* (2013) | 3.0 | 12.0 | 5.0 | 3.0 | 23.0 |
| Segi-Nishida *et al.* (2013) | 2.5 | 11.0 | 5.0 | 2.0 | 22.5 |
| Dryvig *et al.* (2014) | 2.0 | 9.0 | 5.0 | 1.0 | 17.0 |
| Kyeremanteng *et al.* (2014) | 3.0 | 13.0 | 4.0 | 3.0 | 23.0 |
| **Mean** | **2.3** | **10.8** | **4.7** | **2.6** | **20.5** |
