# Supplementary material for: Brain-Derived Neurotrophic Factor and Antidepressive Effect of Electroconvulsive Therapy: Systematic Review and Meta-Analyses of the Preclinical and Clinical Literature
Source: PLoS One. 2015 Nov 3;10(11):e0141564. doi: 10.1371/journal.pone.0141564 (PMC4631320; doi:10.1371/journal.pone.0141564)
Supplement: S2 Table — (DOCX) [file pone.0141564.s002.docx]

| **S2 Table** Quality of the included clinical studies as measured with the Newcastle-Ottawa Scale | | | | |
| --- | --- | --- | --- | --- |
|  | **Selection** [4 max] | **Comparability cases and controls** [2 max] | **Exposure** [3 max] | **Total** [9 max] |
| Bocchio-Chiavetto *et al.* (2006) | 0 | 2 | 0 | 2 |
| Marano *et al.* **(**2006**)** | 0 | 2 | 0 | 2 |
| Okamoto *et al.* **(**2008**)** | 0 | 2 | 0 | 2 |
| Fernandes *et al.* **(**2009**)** | 0 | 1 | 0 | 1 |
| Gronli *et al.*2009 | 1 | 2 | 0 | 3 |
| Piccinni *et al.* **(**2009**)** | 0 | 2 | 0 | 2 |
| Hu *et al.* **(**2010**)** | 2 | 2 | 0 | 4 |
| Gedge *et al.* **(**2012**)** | 0 | 1 | 0 | 1 |
| Haghighi *et al.* ***(***2013**)** | 0 | 1 | 0 | 1 |
| Lin *et al.* **(**2013**)** | 0 | 2 | 0 | 2 |
| Stelzhammer *et al.* **(**2013) | 0 | 1 | 0 | 1 |
| Bilgen *et al.* **(**2014**)** | 0 | 2 | 0 | 2 |
| Bumb *et al.* **(**2014**)** | 1 | 2 | 0 | 3 |
| Kleinmann *et al.* **(**2014**)** | 0 | 2 | 0 | 2 |
| **Mean** | **0.3** | **1.7** | **0.0** | **2.0** |
