# Supplementary material for: Brain-Derived Neurotrophic Factor and Antidepressive Effect of Electroconvulsive Therapy: Systematic Review and Meta-Analyses of the Preclinical and Clinical Literature
Source: PLoS One. 2015 Nov 3;10(11):e0141564. doi: 10.1371/journal.pone.0141564 (PMC4631320; doi:10.1371/journal.pone.0141564)
Supplement: S4 Table — (DOCX) [file pone.0141564.s004.docx]

| **S4 Table.** Brain regions in which BDNF was sampled in the preclinical studies that we included. Brain regions are presented alphabetically. The third column: ‘*Unit of analyses*’ indicates in which ‘analysis’ unit the region was clustered. | | | | |
| --- | --- | --- | --- | --- |
| Tissue sampled | Frequency | | Unit of analyses | References |
| Anterior olfactory nucleus | 1 | | Other | Conti *et al.* (2007) |
| Basolateral amygdaloid nuclei | 1 | | Other | Conti *et al.* (2007) |
| Brainstem | 4 | | Other | Kyeremanteng *et al.* (2014) |
| CA1 | 14 | | Hippocampus | Nibuya *et al.* (1995), Zetterström *et al.* (1998), Chen *et al.* (2001), Conti *et al.* (2007) |
| CA2 | 1 | | Hippocampus | Conti *et al.* (2007) |
| CA3 | 15 | | Hippocampus | Nibuya *et al.* (1995), Zetterström *et al.* (1998), Chen *et al.* (2001), Jacobsen *et al.* (2004), Conti *et al.* (2007) |
| Cerebellum | 8 | | Other | Kyeremanteng *et al.* (2012, 2014) |
| Claustrum | 1 | | Other | Conti *et al.* (2007) |
| Dentate gyrus | 17 | | Hippocampus | Nibuya *et al.* (1995), Zetterström *et al.* (1998), Chen *et al.* (2001), Jacobsen *et al.* (2004), Ryan *et al.* (2013) |
| Dorsal endopiriform nucleus | 1 | | Other | Conti *et al.* (2007) |
| Dorsal hippocampus | 1 | | Hippocampus | Li *et al.* (2007), Gersner *et al.* (2010) |
| Dorsal raphe nucleus | 1 | | Other | Conti *et al.* (2007) |
| Enthorinal cortex | 2 | | Other | Altar *et al.* (2001), Conti *et al.* (2007), |
| Frontal cortex | 29 | | Cortex | Nibuya *et al.* (1995), Altar *et al.* (2001), Angelucci *et al.* (2003), Altar *et al.* (2004), Jacobsen *et al.* (2004), Kyeremanteng *et al.* (2012, 2014) |
| Frontal parietal cortex | 6 | | Cortex | Zetterström *et al.* (1998) |
| Granual layer the dentate gyrus | 1 | | Hippocampus | Conti *et al.* (2007) |
| Granule layer cerebellum | 1 | | Other | Conti *et al.* (2007) |
| Hippocampus | 75 | | Hippocampus | Altar *et al.* (2001), Newton *et al.* (2003), Altar *et al.* (2004), Angelucci *et al.* (2003), Li *et al.* (2006), Ploski *et al.* (2006), Sartorius *et al.* (2009), Kyeremanteng *et al.* (2012, 2014), Luo *et al.* (2012), O’Donovan *et al.* (2012), Segawa *et al.* (2013), Segi-Nishida *et al.* (2013), Dryvig *et al.* (2014) |
| Hypothalamus | 8 | | Other | Kyeremanteng *et al.* (2014) |
| Medial amygdaloid nucleus | 1 | | Other | Conti *et al.* (2007) |
| Medial prefrontal cortex | 3 | | Cortex | Chen *et al.* (2001) |
| Neocortex | 8 | | Cortex | Kyeremanteng *et al.* (2012, 2014) |
| Nucleus accumbens | 1 | | Other | Gersner *et al.* (2010) |
| Occipital cortex | 1 | | Cortex | Angelucci *et al.* (2003) |
| Paraventricular thalamic nucleus | 1 | | Other | Conti *et al.* (2007) |
| **Table SI** *continues on the next page* | | | | |
| **Table SI** *continued* | | | | |
| Parietal cortex | | 1 | Cortex | Nibuya *et al.* (1995), Altar *et al.* (2001) |
| Pirfiform cortex | | 4 | Other | Zetterström *et al.* (1998) |
| Pirfiform gyrus | | 7 | Other | Conti *et al.* (2007) |
| Polymorph layer of the dentate gyrus | | 1 | Hippocampus | Conti *et al.* (2007) |
| Posterior cortical amygdaloid nucleus | | 1 | Other | Conti *et al.* (2007) |
| Prefrontal cortex | | 10 | Cortex | Conti *et al.* (2007), Sartorius *et al.* (2009) |
| Prefrontal cortex layer III | | 1 | Cortex | Conti *et al.* (2007) |
| Peripheral serum | | 13 | Periphery | Sartorius *et al.* (2009), Kyeremanteng *et al.* (2012) |
| Septum | | 1 | Other | Altar *et al.* (2001) |
| Striatum | | 13 | Other | Altar *et al.* (2001), Angelucci *et al.* (2003); Gersner *et al.* (2010), Kyeremanteng *et al.* (2014) |
| Thalamus | | 4 | Other | Kyeremanteng *et al.* (2014) |
| Ventral hippocampus | | 1 | Hippocampus | Gersner *et al.* (2010) |
| Ventral tegmental area | | 1 | Other | Gersner *et al.* (2010) |
| Ventromedial hypothalamic nucleus | | 1 | Other | Conti *et al.* (2007) |
