# Supplementary material for: Brain-Derived Neurotrophic Factor and Antidepressive Effect of Electroconvulsive Therapy: Systematic Review and Meta-Analyses of the Preclinical and Clinical Literature
Source: PLoS One. 2015 Nov 3;10(11):e0141564. doi: 10.1371/journal.pone.0141564 (PMC4631320; doi:10.1371/journal.pone.0141564)
Supplement: S5 Table — (DOCX) [file pone.0141564.s005.docx]

| **S5 Table.** Basic information on the animals that were used in the preclinical studies that were included in our meta-analysis. | | | |
| --- | --- | --- | --- |
| **Study** | **Animal** | **Age in weeks** | **weight** |
| Lindefors *et al.* (1995) | Male Sprague-Dawley rats | N.K. | 150 - 200 gr |
| Nibuya *et al.* (1995) | Male Sprague-Dawley rats | N.K. | 150 - 200 gr |
| Zetterström *et al.* (1998) | Male Sprague-Dawley rats | N.K. | N.K. |
| Chen *et al.* (2001) | Male Sprague-Dawley rats | N.K. | 160 - 180 gr |
| Altar *et al.* (2003) | Male Wistar rats | N.K. | 230 - 250 gr |
| Angelucci *et al.* (2003) | Flinders Sensitive Line rats  Flinders Resistant Line rats | 10 | N.K. |
| Dias *et al.* (2003) | Male Sprague-Dawley rats | N.K. | 200 - 250 gr |
| Newton *et al.* (2003) | Male Sprague-Dawley rats | N.K. | 160 - 180 gr |
| Jacobsen *et al.* (2004) | Male Wistar rats | N.K. | 200 - 300 gr |
| Li *et al.* (2006) | Male Wistar rats | 8 - 10 | N.K. |
| Ploski *et al.* (2006) | Male Sprague-Dawley rats | N.K. | 180 - 220 gr |
| Conti *et al.* (2007) | Male Sprague-Dawley rats | 8 - 10 | N.K. |
| Li *et al.* (2007) | Male Wistar rats | 8 - 10 | 300 - 330 gr |
| Sartorius *et al.* (2009) | Sprague-Dawley rats (no sex specified) | 8 | N.K. |
| Gersner *et al.* (2010) | Sprague-Dawley rats (no sex specified) | 8 | N.K. |
| Kyeremanteng *et al.* (2012) | Male Wistar-Kyoto rats  Male Wistar rats | 7 - 8  7 - 8 | 250 - 350 gr  150 - 250 gr |
| Luo *et al.* (2012) | Male Wistar rats | N.K. | 200 - 240 gr |
| O’Donovan *et al.* (2012) | Male Sprague-Dawley rats | N.K. | 150 - 200 gr |
| Ryan *et al.* (2013) | Male Sprague-Dawley rats | N.K. | 150 - 200 gr |
| Segawa *et al.* (2013) | Male Sprague-Dawley rats | N.K. | 250 - 300 gr |
| Segi-Nishida *et al.* (2013) | Male C57BL/6N mice | 9 - 12 | N.K. |
| Dryvig *et al.* (2014) | Male Sprague-Dawley rats | N.K. | 270 - 350 gr |
| Kyeremanteng *et al.* (2014) | Male Wistar-Kyoto rats  Male Wistar rats | 7 - 8  7 - 8 | 150 - 200 gr  200 - 300 gr |
