# Supplementary material for: Brain-Derived Neurotrophic Factor and Antidepressive Effect of Electroconvulsive Therapy: Systematic Review and Meta-Analyses of the Preclinical and Clinical Literature
Source: PLoS One. 2015 Nov 3;10(11):e0141564. doi: 10.1371/journal.pone.0141564 (PMC4631320; doi:10.1371/journal.pone.0141564)
Supplement: S6 Table — (DOCX) [file pone.0141564.s006.docx]

| **S6 Table.** Basic methodological information on the preclinical studies that were included in our meta-analysis | | | | | |
| --- | --- | --- | --- | --- | --- |
| **Study** | **Method** | **BDNF type** | **Other condition** | **Behavioral tests** | **Sacrificed after** |
| Lindefors *et al.* (1995) | In situ hybridization | BDNF mRNA | None | None | immediately |
| Nibuya *et al.* (1995) | in situ hybridization, Northern blot | BDNF mRNA | None | None | 2 and 18 hours |
| Zetterström *et al.* (1998) | In situ hybridization | BDNF mRNA | None | None | 6, 24, 48 and 504 hours |
| Chen *et al.* (2001) | In situ hybridization | BDNF mRNA | Ketamine add-on conditions | None | 2 hours |
| Altar *et al.* (2003) | ELISA | BDNF mRNA, BDNF protein | None | None | 6, 15, 36, 72, 134, and 240 hours |
| Angelucci *et al.* (2003) | ELISA | BDNF protein | None | None | 24 hours |
| Newton *et al.* (2003) | RT PCR | BDNF RNA | None | None | 2 and 6 hours |
| Jacobsen *et al.* (2004) | ELISA, in situ hybridization | BDNF mRNA | None | None | 18 hours |
| Li *et al.* (2006) | ELISA | BDNF protein | ACTH add-on conditions | Forced swim, locomotor activity, rearing behavior, wet-dog response | 6 hours |
| Ploski *et al.* (2006) | In situ hybridization | BDNF RNA | None | None | 6 hours |
| Conti *et al.* (2007) | In situ hybridization | BDNF mRNA | None | None | 6 hours |
| Li *et al.* (2007) | ELISA | BDNF protein | None | Forced swim test, open-field test: locomotor activity | 24, 48, and 168 hours |
| Sartorius *et al.* (2009) | ELISA | BDNF protein | None | None | 3, 8, 24, 72, 168, and 336 hours |
| Gersner *et al.* (2010) | ELISA | BDNF mRNA | None | Home-cage locomotion, exploration/novelty induced behavior, forced swim test, Morris water maze, sucrose preference test, | 1 hour |
| Kyeremanteng *et al.* (2012) | ELISA | BDNF protein | None | None | 24 and 168 hours |
| Luo *et al.* (2012) | ELISA | BDNF protein | Saline-proponol conditions | Sucrose preference, open-field test | 24 hours |
| O’Donovan *et al.* (2012) | ELISA | BDNF protein | None | Forced swim test, water plus maze | 336 hours |
| Ryan *et al.* (2013) | qRT PCR | BDNF mRNA | None | None | 96 hours |
| Segawa *et al.* (2013) | Western blot,  qRT PCR | BDNF mRNA, protein, Pro-BDNF | None | None | 1, 2, 4, 8, and 24 hours |
| Segi-Nishida *et al.* (2013) | In situ hybridization, qRT PCR | BDNF mRNA | None | Food intake | 2, 4, and 24 hours |
| Dryvig *et al.* (2014) | qRT PCR | BDNF mRNA | None |  | 1, 4, 8, 16, 24, and 48 hours |
| Kyeremanteng *et al.* (2014) | ELISA | BDNF protein | None | Conditioned emotional response, forced swim test, open field test | 24 and 168 hours |
