# Supplementary material for: Brain-Derived Neurotrophic Factor and Antidepressive Effect of Electroconvulsive Therapy: Systematic Review and Meta-Analyses of the Preclinical and Clinical Literature
Source: PLoS One. 2015 Nov 3;10(11):e0141564. doi: 10.1371/journal.pone.0141564 (PMC4631320; doi:10.1371/journal.pone.0141564)
Supplement: S7 Table — (DOCX) [file pone.0141564.s007.docx]

| **S7 Table** Basic information on the patients that were included in the clinical studies that were included in our meta-analysis. | | | | | | | | | |
| --- | --- | --- | --- | --- | --- | --- | --- | --- | --- |
| **Study** | **Characteristics of subjects** | | | **Sub-group** | **N (f/m)** | **ECT** | | **Depression ratings** | |
|  |  |  |  |  |  | **Number of sessions** | **Time point of measurement** |  |  |
|  | **Diagnosis** | **Treatment resistance** | **Age** |  |  |  |  | **Pre-ECT** | **Post-ECT** |
|  |  |  |  |  |  |  |  |  |  |
| Bocchio-Chiavetto *et al.,* (2006) | MDD | yes | 54.0±16.2 | responders | 20 (14/6) | 7 | day after the last ECT | 34.90±7.31# | 8.10 ± 3.24 |
|  |  | yes | 45.0±27.0 | Non- responders | 3 (2/1) |  |  | 29.67±2.01# | 14.00±13.11 |
| Marano *et al.,* (2006) | MDD, BD | NA | 55.9±21.7 | Responders | 13 (3/10) | 7 | day after the 4th ECT | 27.5±6.3 | 7.9±3.5 |
|  |  | NA | 62.0±24.0 | Non-responders | 2 (1/1) |  |  | 29.0±1.4 | 16.0±0 |
| Okamoto *et al.,* (2008) | MDD, BD | yes | 58.6±13.9 | Responders | 12 (6/6) | 12 | 1 week after last ECT | 25.0 ± 8.2* | 10.3± 3.4 |
|  |  | yes | 62.4±15.1 | Non-responders | 6 (3/3) |  |  | 20.6 ± 4.4* | na |
| Fernandes *et al.,* (2009) | MDD, BD | yes | 52.7±15.9 | Responders  (73.33%) | 15 (10/5) | 11.23 | day after the last ECT | 24.15 ± 6.32* | 24.15±6.32* |
| Gronli *et al.,* (2009) | MDD, BD | yes | 70 (40-85) | Responders | 10 (NA) | 12 | immediately prior to discharge | 23.1* | 6.0* |
| Piccinni *et al.,* (2009) | MDD, BD | yes | 47.4±16.7 | Responders | 8 (5/3) | 8.6 | 1 week after last ECT | 24.1 ± 5.3** | 6.8 ± 3.1 |
|  |  | yes | 42.9± 17.9 | Non-responders | 10 (4/6) | 8 |  | 24.1 ± 5.3** | 6.8 ± 3.1 |
| Hu *et al.,* (2010) | MDD | Not known | 43.9±13.8 | Responders | 24 (20/4) | 6 | day after the last ECT | 31.6±4.79 * | 6.5±3.7 |
|  |  | Not known | 23.3±4.2 | Non-responders | 4 (3/1) |  |  | 30.0±3.9* | 18.0 ± 4.1 |
| Gedge *et al.,* (2012) | MDD | yes | 45.7±12.2 | Responders | 5 (2/3) | 12 | 1 week after ECT | 25.6 ±4.93* | 7.8±1.3 |
|  |  | yes | 47.7±7.9 | Non-responders | 6 (5/1) |  |  | 22.17 ±4.40* | 17.83 ± 4.79 |
| **Table SI** *continues on the next page* | | | | | | | | | |
| Haghighi *et al.,* (2013) | MDD | no | 30.7±5.8 | Responders (75%) | 20 (5/15) | 12 | na | 39.35±10.46** | 35.10 ±7.18 |
| Lin *et al.,* (2013) | MDD, BD | yes | 47.4±12.0 | Responders | 48 (38/10) | 9.2 | after the last ECT | 31.5±8.6* | 5.8±3.6 |
|  | MDD, BD | yes | 40.1±8.7 | Non-responders | 7 (6/1) |  |  | 26.1±6.5* | 17.3±8.1 |
| Stelzhammer *et al.,* (2013) | MDD | yes | 49.7**±7.2** | Responders | 3 (3/0) | 12 | 6h after last ECT | 32.3±5.7 | 17.7±10.7 |
|  |  | yes | 58.0±7.7 | Non-responders | 4 (2/2) |  |  | 22.8±4.6 | 21.3 ±6.6 |
| Bilgen *et al.,* (2014) | MDD | Not known | 33.0±5.9 | responders | 30 (19/11) | 5.06 | At the day of response | 30.66±4.11** | 15.73±3.36** |
| Bump *et al.,* 2014 | MDD | Not known | 51.7±13.7 | NA | 20 (10/10) | 11.25 | Varies | 31.2±8.1** | NA |
| Kleinmann *et al.,* 2014 | MDD | yes | 47±16.5 | NA | 11(6/5) | 10 | 24 h after 1st, 4th, 7th and 10th ECT | 34±8.3# | NA |
| Abbreviations: MDD – major depressive disorder; BD – bipolar disorder; “ – HDRS-6; * - HDRS-17; **- HDRS-21; ***- HDRS-24; # - MADRS | | | | | | | | | |
