# Supplementary material for: Brain-Derived Neurotrophic Factor and Antidepressive Effect of Electroconvulsive Therapy: Systematic Review and Meta-Analyses of the Preclinical and Clinical Literature
Source: PLoS One. 2015 Nov 3;10(11):e0141564. doi: 10.1371/journal.pone.0141564 (PMC4631320; doi:10.1371/journal.pone.0141564)
Supplement: S1 Text — (DOCX) [file pone.0141564.s009.docx]

**Quality Assessment**

*Quality of the preclinical studies*

Based on the ARRIVE guidelines for reporting animal research (Kilkenny *et al.,* 2010), two of us (MP and MLM) evaluated the methodological- and reporting quality of the included studies. Overall quality of a study was defined as the number of items that was met by the particular study. Agreement among the raters was excellent (Cohen’s Kappa = 0.95, Standard Error [SE] = 0.1). Overall, the included studies met on average 20 of the 35 quality items (range 14.0 – 25.0). See **Table S4** for the quality of each study (overall, and subdivided by introduction, method, results, and discussion). The quality-score of an individual study was unrelated to the effect-size of the study (*r* = -0.005, *P* = .93). The quality score was related to sample size (*r* = 0.22, *P* = .002) and year of publication (*r* = 0.57, *P* < .0001), indicating that studies that used a larger number of animals and/or that were more recently published were in general of a higher quality.

*Quality of the clinical studies*

Based on the Newcasltle-Ottowa Scale (NOS; Wells *et al,* 2014; The Cochrane Collaboration, 2014), the quality assessment tool that is recommended by the Cochrane collaboration (2014) and the Risk of Bias tool for Longitudinal Studies (RBLS) (Polyakova *et al,* 2014), two of us (MP and MLM) evaluated the quality of the included clinical studies. Overall quality of a study was defined as the number of items that was met by the particular study on each of these scales individually. Agreement among the raters was high (Cohen’s Kappa = 0.90, SE = 0.05 for the NOS and Cohen’s Kappa = 0.76, SE = 0.06 for the RBLS). The included studies met on average 2 of the 8 NOS quality items (range 1 – 4) and 14 of the 35 RBLS items (range 11 – 16). See **Table S5** and **S6** for the NOS and RBLS quality score of the included studies respectively. Both, the NOS and the RBLS quality-score were unrelated to the effect-size of the study (*r* = 0.15, *P* = .61 and *r* = 0.09, *P* = .76 respectively. The NOS quality score was unrelated to sample size (*r* = -0.17, *P* = .56) and year of publication (*r* = 0.10, *P* = .74). The RBLS quality score also was unrelated to year of publication (*r* = -0.16, *P* = *P* = .60) but it was related to sample size (*r* = 0.64, *P* = .02), so that studies that used a larger number of subjects were in general of a higher quality.

**REFERENCES**

Kilkenny C, Browne WJ, Cuthill IC, Emerson M, Altman DG (2010). Improving bioscience research reporting: the ARRIVE guidelines for reporting animal research. *PLoS Biology* **8**: e1000412. doi:10.1371/journal.pbio.1000412

Wells GA, Shea B, O’Connel D. The Newcastle-Ottawa Scale (NOS) for assessing the quality non-randomised studies in meta-analyses. Ottawa Healthcare Institute. http://www.ohri.ca, accessed 11 December 2014

The Cochrane Collaboration Handbookhttp://www.cochrane.org/, accessed 9 November 2014
